# The Predicament and Outlet of Environmental Impact Assessment Mechanism from the Perspective of Risk Society: Taking Japan’s Accident-Type Nuclear Sewage Disposal as a Cut-In

**DOI:** 10.3390/ijerph20053929

**Published:** 2023-02-22

**Authors:** Qiang Zhang, Fang Yang, Yaru Chen

**Affiliations:** 1 School of Law, Qufu Normal University, Rizhao 276800, China; 2 China Institute of Language and Literature, XinJiang University, Urumqi 830046, China; 3 School of Law, East China University of Political Science and Law, Shanghai 201620, China

**Keywords:** accident-type nuclear sewage, environmental impact assessment system, the no-harm principle, risk society

## Abstract

As an important yardstick of social modernization, nuclear technology not only promotes the in-depth development of the national economy but also hangs a sword of Damocles in the field of the risk society. Against the background of the unrest of the nuclear leakage disaster at the Fukushima nuclear power plant, the Japanese government has announced its unilateral decision to discharge nuclear sewage into the sea, undoubtedly putting at least the Pacific Rim countries at huge potential risks. In order to maximize risk reduction and focus on preventive construction in advance, Japan’s measures to discharge accident nuclear sewage into the sea have a legitimate basis for the application of an environmental impact assessment system. At the same time, in the process of operation, there are numerous risk dilemmas, such as lack of safety treatment standards, long follow-up disposal cycle and negative domestic supervision system, which need to be broken through one by one. The effective application of the environmental impact assessment system in the Japanese nuclear accident not only helps to reduce the environmental crisis caused by accidental nuclear effluent discharge to the sea but also has a positive and far-reaching international demonstration significance, which helps to better build a foundation of international trust and preventive guarantee system in advance for the possible accidental nuclear effluent treatment in the future.

## 1. Introduction

As the frequency of human activities increases and the scope of activities expands, the influence of modern human decisions and actions on nature and society itself also increases proportionally, thus making the risk structure gradually evolve from the dominance of natural risks to the dominance of man-made uncertainty risks. For that reason, human society enters a risk society. In the context of risk society, the characteristics of risk diversification are increasingly evident and no country or region is an isolated island; any regional or national risk may have an unpredictable impact on the surrounding regional or even global field. Environmental risks with correlation, trans-temporal and expansive characteristics have become an integral part of modern risk society [1]. To ensure the normal functioning of law and regulation and to make rational decisions after weighing the advantages and disadvantages is the key to avoid various kinds of latent risks in this context, which is the most significant point to obtain relative peace and tranquility in the modern society. However, among the five schemes for the disposal of accident nuclear sewage, such as injection into the earth’s crust, underground burial, conversion into hydride discharge, diluted water into the sea, and water vapor discharge into the atmosphere, Japan has opted for the least costly approach to discharging accident nuclear sewage into the sea [2]. Even a small amount of low-frequency discharge is difficult to ensure harmlessness to other countries and the international public domain in the risk society where the butterfly effect is frequent, not to mention such a huge base and a long cycle. Therefore, Japan’s large-scale and long-term discharge of nuclear sewage into the sea will inevitably create risks and hazards to the surrounding and even global ecological environment. Based on the characteristics of environmental risks involving the whole body, in order to maximize the reduction in environmental risks and damage caused, thorough risk prevention and control should be carried out in all aspects of the nuclear effluent discharge operation. Compared with the problem of determining and assuming responsibility afterwards, the preventive stage of environmental protection at the source is particularly important. Thus, it is necessary and feasible to take the environmental impact assessment system as an important means to deal with the environmental risks which are frequently encountered in the modern risk society, such as the treatment of nuclear sewage by accident.

In the early 20th century, nuclear science and technology achieved rapid development and major breakthroughs in both theoretical and experimental research [3]. The nuclear energy provides human society with a tremendous amount of energy unmatched by other energy sources nowadays. According to the statistics of the International Atomic Energy Agency (IAEA), by the end of June 2022, more than 30 countries or regions in the world have built nuclear power plants, with 440 operating units and 53 nuclear power units under construction. Among them, a large number of nuclear power plants are built along the coast based on the consideration of nuclear power unit cooling, waste heat emission emergency, and so on. There is a huge risk crisis behind these magnificent nuclear power plants. As early as 1986, some scholars presciently argued that any discharge of radioactive waste into the ocean should be banned [4]. If they all follow Japan’s example of discharging accidental nuclear sewage into the sea, once the risks are realized, the disaster to the world will be inestimable and devastating. Under the premise that the society cannot get rid of the dependence on nuclear energy, the international community pays increasing attention to nuclear safety management [5].

Although the Fukushima nuclear accident is a super design reference accident caused by earthquake and tsunami, it indeed belongs to a small probability event from the perspective of probability safety; however, the damage consequences are incalculable [6]. Therefore, in the risk society with the irresistible development of nuclear technology, while enjoying the technological dividend brought by nuclear power, it is of great significance to timely consider diversified kinds of risks that may be brought by its application and to establish and improve the prevention and early warning system. Whether it is based on the considerations of economic benefits, environmental ethics or damage minimization, compared with the much longer and more ambiguous process of determining and assigning responsibility during and after the event in the international environmental law, choosing to focus on the preventive construction in advance is obviously more operable and practical. An environmental impact assessment system was originally proposed by the domestic law of the United States, and its concept was formally established at the International Environmental Quality Assessment Academic Conference held in Canada in 1964. With the increasing number and difficulty of cross-border environmental risks in the risk society, the environmental impact assessment system has been increasingly valued by countries around the world. The environmental impact assessment system aims to reduce the significant negative transboundary environmental risks that may be caused by human activities, and it takes the countries of source of behavior and other countries that may suffer from environmental risks, as well as relevant international organizations and institutions as multiple subjects, to analyze and predict potential environmental risks, to provide decision makers with relevant information on possible environmental risks arising from the formulation of activities and to help improve the scheme of activities.

As an essential legal means in the prevention stage of environmental protection, the environmental impact assessment system plays an increasingly constructive role in dealing with such events that may cause the transboundary environmental impact in the ever-changing international community. For activities that may cause major cross-border environmental risks, such as accidental nuclear sewage discharge, before the official implementation of the activity in the country of origin, the potential cross-border environmental impacts should be predicted, assessed and analyzed in accordance with scientific and consultative environmental protection standards, and diversified public participation modes such as hearings should be adopted that can directly reflect the public interest. In the whole process of environmental impact assessment system implementation, a sound supervision system ought to be built. Therefore, taking multidimensional, appropriate and scientific preventive measures against potential environmental risks can not only effectively reduce or eliminate the significant adverse impact that the activity may have on the environment but also help to consolidate the trust foundation between the affected countries and the people on the government, consolidate the authoritative image of the government internally and strengthen international cooperation externally.

## 2. The Justification Basis of the Application of Environmental Impact Assessment System

### 2.1. Obligation Basis

Japan’s unilateral announcement of the decision and action of dumping accidental nuclear sewage into the sea not only violates a number of international environmental protection obligations but also violates relevant international laws in many ways [7], such as the United Nations Convention on the Law of the Sea and the Convention on the Prevention of Marine Pollution by Dumping of Wastes and Other Matter. In a nutshell, it mainly includes the following six aspects of obligation violations on the basis of deviating from the declarative provisions on the protection and preservation of the marine environment. (1) To begin with, it did not use the most feasible method to treat accident nuclear sewage in accordance with its domestic capacity, and it chose the one with the lowest cost, the greatest damage to the environment and the strongest impact on foreign affairs among the five schemes, which did not match its treatment capacity appropriately [8]. (2) In the second place, by laying tunnels along the coast of the inland sea, millions of tons of nuclear sewage will be dumped from the land sources, which contains a large amount of toxic, harmful and unwholesome substances that are very difficult to filter and purify. (3) The third aspect is that for the index detection of accidental nuclear sewage planned to discharge into the sea, Japan has not adopted the internationally recognized scientific method. Not only the sources and basis, but also the methods and results of sampling are not fully open. For that reason, it is difficult to demonstrate the scientific basis of the data results. In addition, the channels and extent of the international community’s monitoring of pollutant discharge activities are limited. (4) The participation in decision-making and actions of accidental nuclear sewage discharge in Japan is grossly inadequate and deficient, even if Japan requests the International Atomic Energy Agency to provide technical support and supervision. However, there is still a lack of discourse decision on the application of safety standards and the implementation of safety advisory review [9]. (5) Fifth, Japan’s unilateral actions do not focus on international cooperation among neighboring affected countries and lack of the promotion of regional linkage. Although numerous actions have been taken through the International Atomic Energy Agency, a competent international organization, efforts must be made to formulate not only the global and regional rules, but also the standards and recommended methods and relative procedures [8]. (6) Last but not least, Japan’s unilateral accident nuclear sewage discharge decision was made and even partially dumped without the permission of the competent authorities of the surrounding countries that may be affected [8].

As an advocate of environmental diplomacy, Japan has a contradictory dual orientation on the issue of marine discharge of nuclear sewage. On the one hand, in order to effectively regulate the discharge of waste materials into the sea by other countries, it zealously advocated the signing of the Convention on the Prevention of Marine Pollution by Dumping of Wastes and Other Matter in 1996, requiring that wastes with “credible suspicion” be strictly prohibited from dumping into the sea. On the other hand, in the event of the Fukushima nuclear accident, the Japanese government declared that it would “minimize the flow of radioactive material into the sea” but chose the lowest-cost way to dump it into the sea among the five disposal options. In order to circumvent the obligations of the Convention on the Prevention of Marine Pollution by Dumping of Wastes and Other Matter, Japan even abandoned the cheaper plan to dump contaminated nuclear water into the ocean via ships and switched to discharge the accidental nuclear sewage in the inland waters of Japan in the form of undersea tunnels. Nevertheless, such a measure is still subject to the restrictions on emissions from land-based sources as provided for in article 194 of the United Nations Convention on the Law of the Sea.

Japan’s unilateral decision on the discharge of accidental nuclear sewage into the sea and the previous unauthorized dumping constitute illegal acts in international law, which not only shows the true face of irresponsibility under the mask of its environmental diplomacy but also reveals that Japan should bear procedural prudential obligations in the event of violation of substantive obligations [10]. In order to achieve the goal of controlling and minimizing the environmental damage to the surrounding countries and the international public domain as much as possible, the environmental impact assessment system should be fully applied to establish the safety standard of pollution discharge, build a sound supervision system, predict the possible consequences and design numerous alternative schemes by strengthening international cooperation and interregional linkage in advance.

### 2.2. Principle Basis

#### 2.2.1. The No-Harm Principle

As the cornerstone of international environmental law and the basic rule of various environmental treaties and declarations, the No-Harm Principle, the “golden principle” [11], was first established by the United States in 1941 in the Terrell smelter arbitration case of Canada. It was stipulated as an authoritative rule in the 1972 Stockholm Declaration on the Human Environment. The No-Harm Principle is consistent with the social value system of safety pursuit [12]. The basic connotation of this principle consists of two opposite pulling forces. It does not allow a country to wantonly carry out environmental development activities in its field regardless of foreign influence nor does it allow other countries to wantonly interfere in development activities within the sovereign jurisdiction of a country. On the premise of respecting national sovereignty, the State, as the subject responsible for the cross-border impact caused by development activities, should bear the responsibility of prevention, notification, consultation and negotiation [13].

In the specific situation of accident-type nuclear sewage discharge in Japan, the application of the No-Harm Principle is mainly reflected in that Japan enjoys relatively adequate autonomy in the treatment of its own nuclear sewage, and other countries and institutions have no right to interfere and restrict it. However, this full autonomy is based on the premise that the approach, the result and the subsequent impact of nuclear sewage disposal in Japan are not foreign-related. Otherwise, other countries and relevant international organizations have the legitimate rights and interests to require Japan to make corresponding explanations and plans for rectification and reform.

In the horizontal view, according to the International Nuclear event rating scale (INES), the Chernobyl nuclear accident in the former Soviet Union in 1986, which was also rated as level 7, chose to seal the accident nuclear power plant with sarcophagus. Although the causes of nuclear accidents are different, there are still some contents worth comparison and reflection in the two highest level nuclear accidents, which are mainly reflected in the following three aspects: (1) the number of direct deaths caused by nuclear accidents: 53 people died directly in Chernobyl and 0 people died in Fukushima; (2) the extent of the exclusion zone after the accident: the exclusion zone of the Chernobyl nuclear power plant is 30 kilometers, and that of the Fukushima nuclear power plant is 20 kilometers; (3) estimated leakage amount: in addition to the amount of xenon-133 in the Fukushima nuclear accident, which was nearly twice that of Chernobyl, the amount of radioactive substances such as iodine-131 and strontium-89 in the Fukushima nuclear accident was much lower than the latter. Therefore, it can be known from the comparison of data that in terms of the direct damage degree of nuclear accidents, the environmental risk degree of the Chernobyl nuclear accident is higher, but the former Soviet Union government has taken the desperate determination to bear the responsibility to keep the environmental risk within its own domain as much as possible. Through the continuous reinforcement, maintenance and updated storage construction, the impact of the Chernobyl nuclear accident was locked in the domestic domain as far as possible, so as to maximize the damage to the environment of other countries and the public domain.

Unlike this kind of nuclear accident waste disposal, which completely controls development activities and enjoys a high degree of autonomy, Japan’s measures to discharge accident nuclear sewage into the sea as a public domain are bound to cause damage to its territorial sea environment while profoundly affecting at least the environment of the Pacific Rim countries and the international public area. Such transboundary development activities, which infringe upon the public interests of other countries and the international community, are subject to the natural regulation of the No-Harm Principle; measures ought to be taken, including the formulation of preventive measures, prevention and reduction, and detection of widespread environmental impacts [14], to control and reduce the extraterritorial spread of environmental damage as much as possible. First, measures to mitigate environmental degradation should not be delayed. Environmental risks also have uncertainties in the probability of occurrence and the scope of damage. However, when certain scientific evidence can prove that the possibility of occurrence of damage is high, measures should be taken even if the evidence is insufficient. Secondly, take cost-effective preventive measures according to the actual situation of environmental risks and the overall situation of the host country to achieve a balance between economic development and environmental protection; Finally, the public’s right to participate in the monitoring and investigation data of environmental risk assessment should be guaranteed through hearings and other forms to safeguard the legitimate rights and interests of the public.

#### 2.2.2. Principle of Intergenerational Equity

The continuation of a species is the instinctive demand of human beings, and the consideration of the interests of future generations is also the conscience of civilized human beings [15]. As one of the basic principles of the ecological law, the principle of intergenerational equity is the theme of sustainable development concerned by the United Nations Conference on Environment and Development in 1992 [16]. The principle of intergenerational equity is based on the rational prediction of the survival crisis that may be encountered by future generations, and it refers to the interests of the diachronic human community of contemporary and future generations [17]. It not only has the guiding significance to the environmental impact assessment system with preventive attribute from the macro-system operation level but also has the minimum restriction effect on the accident nuclear sewage discharge behavior at the specific operation level. From the perspective of nuclear power-assisted risk society, the decision making between countries makes the intergenerational relationship between the present and the future generations of the global village closer. The perspective provided by the principle of intergenerational equity based on the overall position of preventing the world ecological crisis rather than narrow individual interests is worthy of careful consideration when countries make decisions.

In the specific situation of accident nuclear sewage discharge in Japan, the application of the principle of intergenerational equity should be based on a battery of scientific logical argumentation and the judgments of legal and social value [18]. (1) On the one hand, at the logical level of scientific argumentation, in order to keep the damage to the sustainable environmental rights and interests at home and abroad and to future generations under control, the sea discharge behavior of accident nuclear sewage ought to be supported by scientific decision making and technology beforehand, including the establishment of the bottom line standard for safety treatment in advance, the clear purification capacity of the purification and disposal device for radioactive and other polluting substances, and on this basis to continuously improve the existing technology in the above process. (2) On the other hand, at the level of judgment and choice of both legal and social value, as a legal measure to deal with the global environmental crisis, the principle of intergenerational equity shows concern for the basic ethical values of society in a good deal of international environmental conventions. In its first entry, the Joint Convention on the Safety of Spent Fuel Management and on the Safety of Radiation Waste Management stipulates that preventive measures for the disposal of radioactive waste should fully take into account the sustainable development of future generations on the basis of meeting the needs and aspirations of contemporary people [19]. And in the general safety requirements of radioactive waste stipulated in Article 11, it is clearly pointed out that it should be based on the scientific basis to avoid excessive burden on the future generations.

Nuclear safety is the supreme principle of the use of nuclear energy. The whole aspect and whole process guarantee of nuclear safety in each nuclear power country is related to the sustainable environmental safety framework of all nuclear power countries and even the global countries [9]. As a measure of post-disposal of nuclear safety, the discharge of accidental nuclear sewage in Japan is not only related to the synchronic interests of contemporary people but also closely related to the diachronic sustainable development rights and interests of the future generations. Therefore, the preventive work such as environmental impact assessment is of great and far-reaching significance in the operation of accident nuclear sewage discharge.

## 3. The Risk Dilemma of the Application of the Environmental Impact Assessment System

### 3.1. Lack of Safety Handling Standards

The environmental impact assessment system constructs the obligation system with rationalization as the standard and judges the realization of the obligation by referring to the results of the objective standard, but the core of the problem lies in the selection and establishment of the judgment yardstick. In the specific context of nuclear sewage discharge from the accident in Japan, there are three problems in the determination of this scale as hereunder mentioned.

The first problem to bear the brunt is, there is no international precedent for Japan to discharge accidental nuclear sewage into the sea. In the macro view of the rationalization standard of environmental assessment, it is extremely untoward to find the reference point from the vertical precedent dimension and to define the direction from the horizontal national treatment plan dimension. The second issue that ensues is Japanese officials’ confusion about the concept of accidental nuclear sewage. It is universally acknowledged that there is a fundamental difference in the quality and quantity between the accident nuclear sewage in Japan and the nuclear sewage produced by the daily operation of nuclear power plants. The accident nuclear sewage discharged into the ocean is not the same as the industrial sewage produced by other nuclear power plants based on controllable leakage and periodic cleaning of equipment, and the damage to the marine environmental system caused by the radioactivity and heavy metals contained in the former is incalculable. Even if the Japanese government and Tokyo Electric Power Co repeatedly claim that the Advanced Liquid Processing System (ALPS) treats nuclear sewage to meet the standard before discharge, it is certain that the treated nuclear sewage is safe and reliable. The crux of the problem is that although the Japanese government claims to filter out 62 radioactive substances other than tritium, the concentration of tritium in water is diluted to less than 1/40 of the national tritium emission standard for nuclear power plant wastewater in Japan, that is, tritium activity per liter of water is 60,000 becquerels [20]. However, this intuitive visibility of “safety and reliability” is only for the fact that the “tritium” elements have met the discharge standards. However, it is not known whether the purification and treatment of other radioactive substances such as strontium-90 and carbon-14 in accident nuclear sewage has been filtered out as officially claimed.

As a preconceived crisis, the Fukushima nuclear accident is an inflection point from quantitative change to qualitative change in the construction of nuclear safety in Japan [21]. Especially in the absence of this kind of safety treatment standard, the processing procedure and result evaluation of sampling and testing not only lack an open and transparent data analysis process but are also at a loss to measure the feasibility of emission. In the treated water sampled on 28 July 2022, the activity of strontium-90 was 93 becquerels per liter (the Japanese national standard value was 30 becquerels), but on August 4, the activity of strontium-90 in the treated water was changed to 2.7 becquerels. In view of such a wide gap, Tokyo Electric Power Co did not give a clear reason or a convincing explanation, only speculating that the problem may lie in the second half of the process. Moreover, Japan’s official resolution does not reflect the recommendations of the international peer group in its review of the “Mid-and-Long-Term Roadmap towards the Decommissioning of TEPCO’s Fukushima Daiichi Nuclear Power Station Units 1–4.” [22] The test results of the absence of scientific demonstration are not only difficult to convince the international community but also further shake the legitimate scientific basis of nuclear sewage discharge behavior.

### 3.2. The Follow-Up Disposal Period Is Long

As shown in Table 1, not only representatives of relevant industries in Japan expressed strong opposition to the Japanese government’s action, but also the neighboring countries that may suffer from high environmental risks held firm opposition positions. However, Japan has decided to release contaminated water into the sea despite opposition from many parties. The Japanese government needs to bear corresponding legal responsibility for the damage to persons, property and the environment that crosses national boundaries when it engages in acts not prohibited by international law [23]. It is the natural responsibility of the Japanese government to scientifically and reasonably plan the subsequent long disposal cycle.

Although there are differences between the Chernobyl nuclear accident and the Fukushima nuclear accident in terms of disposal methods, the disposal cycle of the Chernobyl nuclear accident can provide a certain reference for the disposal cycle of the accident-type nuclear wastewater in Japan. Completed in 1986 at a cost of $18 billion, Sarcophagus 1 began to malfunction in 2008. The construction of Sarcophagus II was completed in 2016 at a cost of 935 million euros and is expected to last 100 years. Thus, a century-long safety bolt has been added for the nuclear accident, which has temporarily put an end to the 30-year disposal work. However, for Japan, there are more factors to be considered in the process of disposal. The self-purification capacity of the ocean is limited, especially in the face of such a huge base and severe pollution index of accidental nuclear sewage, and it is impossible to make a hasty decision to discharge the sea. Although the ocean can purify a series of radioactive substances and contaminated substances in the accident-type nuclear wastewater by physical, chemical and biological means, on the one hand, unlike the general industrial nuclear wastewater, many radioactive substances in the accident-type nuclear wastewater are difficult to purify in a short time or even cannot be purified. On the other hand, the self-purification capacity of the ocean is not unlimited, among which the most important physical purification method will further spread the pollutants in the accident-type nuclear wastewater to the sea, resulting in cross-border environmental risks. The weight base of accident nuclear sewage and the difficulty of purification are not the only reasons for the long disposal cycle. Up to now, the Fukushima nuclear power plant has been exposed in the public view of nuclear waste containers that have leaked as many as three times, and this does not include the leakage that it deliberately concealed [24]. The towering million-ton nuclear sewage storage tanks standing around the Fukushima nuclear power plant have the possibility of leakage; once the terrible risk is realized, the subsequent transfer, repair, treatment and other processes are of great complexity. What is more, these million-ton storage tanks are not actually the upper limit of nuclear sewage produced by the Fukushima nuclear power plant. The amount of groundwater and the pollution of rain caused by typhoons or rainy seasons in Fukushima as a coastal area is also increasing with each passing day. Since the discovery of radioactive wastewater leakage at the Fukushima nuclear power plant and Japan’s approval of the discharge of low-level radioactive contaminated water, the migration and diffusion of radioactive materials through the sea and the prediction of radioactive consequences have become a focus [25]. For this reason, the long cycle of purification and disposal of accident nuclear sewage caused by the above reasons increases the overall difficulty of environmental impact assessment.

### 3.3. The Domestic Oversight System Is Negative

After World War II, Japan began the policy of denuclearization but focused on developing the commercial nuclear technology [26]. In the early stages of construction, in order to vigorously promote nuclear power equipment and technology, Japanese officials used the excuse of power shortages in emerging countries to use nuclear power as a driver of their economic development strategy [27]. However, in the wake of the Fukushima nuclear accident, compared with the waves of public opposition, the Japanese government holds a more unified support position for the accidental discharge of nuclear sewage into the sea. In order to allay the public’s concerns about various kinds of pollution, the authorities even carried out the act of beautifying the discharge of pollutants, such as using the radioactive isotope “tritium” as the mascot in the propaganda of nuclear sewage discharge. It is even mentioned several times in the diplomatic Blue Book that the Fukushima nuclear accident has damaged Japan’s reputation as an attempt to create a national image of the victim [28].

In addition, Japan’s official statement on the discharge of nuclear sewage into the sea contradicts its treatment measures, and its credibility is greatly reduced. For example, since Tokyo Electric Power Co announced and implemented the nuclear sewage discharge plan on 4 April 2011, it has discharged a total of 10,393 tons of nuclear sewage up to 10 April of the same year. After triggering strong protests at home and abroad, the former chairman of Tokyo Electric Power Co, Sheng Minamata, made an overt permanent commitment not to discharge nuclear sewage into the sea. However, on 13 April 2021, Japan made an astounding decision to discharge millions of tons of nuclear sewage into the sea [29].

The Fukushima accident shattered public confidence in the safety of nuclear energy and had a huge impact on the recovering nuclear power industry worldwide [30]. Therefore, the public’s acceptance of nuclear energy has a negative impact. Psychologically, they are more anxious about nuclear safety, and some of them even have anti-nuclear sentiment [31]. In the face of the huge national power, the voice of the people will inevitably be weakened. What is more, under this official system of beautifying the wanton discharge of nuclear sewage into the sea, it is even more difficult to carry out open, procedural, sound and effective domestic supervision of the nuclear sewage treatment and discharge process with such a huge base and a long cycle. The request for the Japanese government to conduct good maritime governance, whether through effective consultation or timely notification, is out of the human community’s obligation to protect the ecological environment and the hope that the risk of nuclear pollution can be effectively prevented [32].

## 4. The Perfect Path to the Application of Environmental Impact Assessment System

### 4.1. Establish the Bottom Line Treatment Standard Scientifically

Every major nuclear accident has, to a certain extent, promoted the technological level of nuclear power to leap forward once again, prompting the international community to significantly improve the concept of nuclear safety and nuclear safety standards [33]. The establishment of the standard for the safe treatment of accidental nuclear sewage is the first step to steadily promote the application of the environmental impact assessment system. The determination of this standard should be aimed at maximizing the infringement and danger caused by discharge to the surrounding countries and the international public domain while respecting Japan’s sovereignty and independence. Under this dual protection purpose, based on the realistic consideration of improving the possibility of operation, the lowest but not too high bottom line standard ought to be established for the treatment of accidental nuclear sewage. The establishment of the minimum standard is supposed to be based at least on the following scientific basis, justifiable normative basis and procedural basis.

Scientific foundation: At the present stage, the Advanced Liquid Processing System is the main force for the purification of accident nuclear sewage in Japan. However, due to the incomplete openness of purification data and the singleness of data sources, whether the purification degree of tritium by the Advanced Liquid Processing System can meet the emission standards as claimed by Japanese officials, and whether strontium-90, carbon-14 and other radioactive materials can be purified synchronously are of great ambiguity. Although it may involve internal information in Japan, matters such as purification results, which are closely related to the environment of neighboring countries and the international public domain, should be made more public. On this basis, the scientific treatment and discharge basis of accident nuclear sewage is established by comparing the discharge index of domestic dischargeable sewage in Japan, the discharge index of nuclear sewage produced by daily operation of nuclear power plant, and the standards recognized by the international community and then taking the lowest level that can be achieved as the required standard.

Normative basis: The bottom line for the safe treatment of accidental nuclear sewage should be formulated by further perfecting, supplementing and refining domestic legislation under the framework of abiding by the provisions of correlative international conventions. Comply with the planning and controlled requirements of the Joint Convention on the Safety of Spent Fuel Management and on the Safety of Radiation Waste Management [19]. On the basis of defining the total amount of accident nuclear sewage, it is scientifically divided according to its sampling value and marine dynamic purification capacity, and the components are discharged in batches and orderly. Treat the opinions and suggestions of the International Energy Agency scientifically and at the same time promote regional governance linkage through consultation on multilateral treaties among neighboring countries that may have environmental risk impacts.

Procedural basis: For the discharge of accident nuclear sewage, on the premise of meeting the above-mentioned scientific and normative basis, it is also necessary to further follow the discharge standards established by the International Energy Agency and the domestic government. In addition, in the view of the foreign nature of the discharge and the seriousness of nuclear pollution, the sampling and testing data ought to be made public before discharge, and the results of purification treatment and the expected impact on the environment should be evaluated and assessed. Accept the inquiry and supervision of the domestic public and the international community in the form of hearings and press conferences so as to reduce the harm of accident nuclear sewage to the environment as much as possible. In addition, attention should be paid to the convenience of data opening to promote the conversion of professional data into content that the public can understand [34].

### 4.2. Follow Up the Progress of the Follow-Up Process Transparently

Although the Japanese government has repeatedly claimed that the nuclear wastewater to be discharged from the accident meets safety standards, even if it does, it cannot guarantee that such a long-term and massive discharge will not cause damage to the global environment [35]. In view of the long post-treatment work of Japan’s accident-type nuclear sewage, including purification and discharge, the International Atomic Energy Agency has officially launched a multi-year review of Japan’s discharge of nuclear sewage into the sea. The review will be carried out throughout the whole cycle before, during and after the event [36]. Although to a certain extent, this move has allayed the concerns of the Japanese public and the international community about Japan’s previous improper unilateral behavior, such as unauthorized emissions, it is not a panacea. The construction of a trust mechanism needs to be based on the openness and transparency of information and procedures and the full participation of multiple agents.

On the one hand, in order to further broaden the coverage of trust, the countries of the specific region that may be affected by the most closely related interests can be taken as the main participants to participate in the follow-up of accident-type nuclear sewage disposal in an orderly manner under the coordination and leadership of the International Atomic Energy Agency. On the other hand, under the framework of only the formal participation of the International Atomic Energy Agency, there is a lack of subsequent efforts by the international community on the data and information published by Japanese officials and the decision-making process. Japan needs to further fill the gap of information asymmetry and improve the decision-making process on the premise of safeguarding its sovereignty and independence so as to avoid the crisis of trust caused by unauthorized discharge and leakage.

### 4.3. Construct a Pluralistic Supervision System Cooperatively

The Japanese government’s plan for discharging the nuclear sewage into the sea obviously has problems such as short-sighted thinking and utilitarianism while ignoring potential risks as well as incomplete information disclosure and inadequate communication [37]. For the current situation of negative, single and even reverse beautification of accidental nuclear sewage discharge in Japan’s domestic supervision system, the solution is supposed to adhere to the domestic and foreign two-pronged approach under the technical and procedural supervision framework of the International Atomic Energy Agency to build a positive and diversified supervision system.

On the one hand, public participation is one of the most crucial elements in an environmental impact assessment system, which can guarantee the democracy of administrative decision making [38]. For that reason, a two-way domestic supervision framework should be constructed, which includes not only the public’s export-oriented supervision over the relevant departments involved in the whole process of sewage discharge, including publicity, but also the introverted supervision of the relevant departments by special government supervision agencies. For the public-oriented export-oriented supervision system, first of all, it is necessary to protect the public’s formal right to be informed, disclose information on the basis of the truth of information, and correctly characterize the behavior of accidental nuclear sewage discharge into the sea. The government should avoid further propaganda and beautification and other deceptive acts of “brainwashing” directed at the public. In the second place, it is necessary to protect the public’s substantive right to be informed, by holding periodical hearings and press conferences, to give a general explanation of the meaning, possible result orientation and scope of influence of the professional and scientific knowledge terms and data involved., so as to consolidate the substantive ability foundation of the domestic public to carry out effective supervision. Last but not least, the public’s right of inquiry also needs to be protected formally. When the public has doubts about the official data, publicity and other related contents, and supervises the legitimacy and legality of the behavior, the departments concerned ought to invite relevant professionals to answer the questions and give reasonable explanations. Through the above social supervision, the alienation of state power in the process of nuclear sewage discharge can be effectively prevented so as to make it more in line with the needs of social development and safeguard the interests of the public [39].

On the other hand, tamp the normative foundation of a foreign supervision path. For transboundary ecological and environmental issues, relevant countries should break environmental barriers and carry out full regional cooperation based on regional and even global environmental interests to increase global social welfare [40]. In view of the balance between respecting Japan’s national sovereignty independence and derogating from environmental violations to neighboring countries and the international public under the framework of the principle of preventing environmental damage, relying solely on moral constraints and determining the behavior boundaries of both parties does not have the effectiveness of performing obligations and is difficult to provide legitimate support for subsequent responsibility distribution and commitment. Therefore, it is not only necessary to establish international supervision over the discharge of sewage from Japan’s nuclear accident through international treaties as well as the normative basis for Japan to maintain its own national sovereignty and prevent excessive interference from other countries. It is also an important part of the foreign supervision system, which is mutually beneficial to both sides. The Pacific Rim countries that are the first victimized group to be affected by pollutant discharge can now conclude agreements to conduct normative pilot projects with specific geographical areas as the threshold for entry and common interests as the link. A normative supervision system is based on the international agreement on the Prevention of Marine pollution by the dumping of wastes and other substances. It can orderly and effectively strengthen international cooperation in accordance with the statute, achieve information sharing, supervise and reduce the damage to the environment in the region, and at the same time avoid improper interference to Japan’s national sovereignty as far as possible.

## 5. Conclusions

In the perspective of risk society, transboundary environmental risks are also frequent due to human factors such as scientific and technological progress and decision making. The rapid development of nuclear technology makes nuclear energy not only provides convenience for production and life in modern society but is also an important manifestation of a country’s comprehensive national strength. However, the rapid development of nuclear energy has also brought huge risks and hidden dangers to human society, especially the ecological environment. The environmental risks caused by nuclear leakage show a trend in direct proportion to the booming development of nuclear energy. In this environmental risk perspective, the environmental impact assessment system, which is a positive legal mechanism of environmental protection and prevention stage, has both environmental protection and economic significance. The application of the environmental impact assessment system has a universal international demonstration role in reducing the environmental risks caused by the disposal of accident-type nuclear sewage in Japan. It not only promotes the institutional construction of the environmental impact assessment system but also strengthens the trust foundation of affected countries and citizens on the government, strengthens the authoritative image of the government internally and strengthens international cooperation externally.

## Figures and Tables

**Table 1:** Attitudes of related industries and neighboring countries that may be affected.

Number	Nation/Organization	Attitude
1	Japan National Federation of Fisheries Cooperative Associations	Strongly protested and will continue to oppose it in the future.
2	Philippines	Strongly condemns and calls for joint action to boycott Japan’s plan to discharge accidental nuclear sewage into the sea.
3	China	Expresses grave concern over Japan’s plan and strongly urges the Japanese side to recognize its responsibilities and fulfill its international obligations.
4	South Korea	Strongly protested and will actively explore applying for interim measures or filing a complaint with the International Tribunal for the Law of the Sea.
5	United States	Supports the Japanese government’s decision, Japan’s plan is in line with internationally recognized nuclear safety standards.
6	Greenpeace	It is “unfair” for the Japanese government to discharge nuclear waste water into the sea, which may damage human DNA.
7	International Atomic Energy Agency	Supports the discharge of the accumulated treatment water from the Fukushima Daiichi nuclear power plant into the sea, which is in line with international practice.

## Data Availability

No new data were created or analyzed in this study. Data sharing is not applicable to this article.
